# Long-term efficacy and safety of sapropterin in patients who initiated sapropterin at < 4 years of age with phenylketonuria: results of the 3-year extension of the SPARK open-label, multicentre, randomised phase IIIb trial

**DOI:** 10.1186/s13023-021-01968-1

**Published:** 2021-08-03

**Authors:** Ania C. Muntau, Alberto Burlina, François Eyskens, Peter Freisinger, Vincenzo Leuzzi, Hatice Serap Sivri, Gwendolyn Gramer, Renata Pazdírková, Maureen Cleary, Amelia S. Lotz-Havla, Paul Lane, Ignacio Alvarez, Frank Rutsch

**Affiliations:** 1 University Children’s Hospital, University Medical Center Hamburg-Eppendorf, Martinistrasse 52, 20246 Hamburg, Germany; 2 University Hospital, Padua, Italy; 3 Universitair Ziekenhuis Antwerpen, Antwerp, Belgium; 4 Children’s Hospital Kreiskliniken, Reutlingen, Germany; 5 Universita La Sapienza, Rome, Italy; 6 Hacettepe University School of Medicine, Ankara, Turkey; 7 Division for Neuropaediatrics and Metabolic Medicine, Centre for Paediatric and Adolescent Medicine, University Hospital Heidelberg, Heidelberg, Germany; 8 Department of Children and Adolescents, Third Faculty of Medicine, Charles University, Prague, Czech Republic; 9 Great Ormond Street Hospital, London, UK; 10 Dr. Von Hauner Children’s Hospital, Munich, Germany; 11 BioMarin Europe Ltd., London, UK; 12 Muenster University Children’s Hospital, Muenster, Germany

**Keywords:** Hyperphenylalaninaemia, Phenylketonuria, Infants, Therapy recommendations, Sapropterin dihydrochloride

## Abstract

**Background:**

During the initial 26-week SPARK (Safety Paediatric efficAcy phaRmacokinetic with Kuvan®) study, addition of sapropterin dihydrochloride (Kuvan®; a synthetic formulation of the natural cofactor for phenylalanine hydroxylase, tetrahydrobiopterin; BH_4_), to a phenylalanine (Phe)-restricted diet, led to a significant improvement in Phe tolerance versus a Phe-restricted diet alone in patients aged 0–4 years with BH_4_-responsive phenylketonuria (PKU) or mild hyperphenylalaninaemia (HPA). Based on these results, the approved indication for sapropterin in Europe was expanded to include patients < 4 years of age. Herein, we present results of the SPARK extension study (NCT01376908), evaluating the long-term safety, dietary Phe tolerance, blood Phe concentrations and neurodevelopmental outcomes in patients < 4 years of age at randomisation, over an additional 36 months of treatment with sapropterin.

**Results:**

All 51 patients who completed the 26-week SPARK study period entered the extension period. Patients who were previously treated with a Phe-restricted diet only (‘sapropterin extension’ group; n = 26), were initiated on sapropterin at 10 mg/kg/day, which could be increased up to 20 mg/kg/day. Patients previously treated with sapropterin plus Phe-restricted diet, remained on this regimen in the extension period (‘sapropterin continuous’ group; n = 25). Dietary Phe tolerance increased significantly at the end of the study versus baseline (week 0), by 38.7 mg/kg/day in the ‘sapropterin continuous’ group (95% CI 28.9, 48.6; *p* < 0.0001). In the ‘sapropterin extension’ group, a less pronounced effect was observed, with significant differences versus baseline (week 27) only observed between months 9 and 21; dietary Phe tolerance at the end of study increased by 5.5 mg/kg/day versus baseline (95% CI − 2.8, 13.8; *p* = 0.1929). Patients in both groups had normal neuromotor development and growth parameters.

**Conclusions:**

Long-term treatment with sapropterin plus a Phe-restricted diet in patients who initiated sapropterin at < 4 years of age with BH_4_-responsive PKU or mild HPA maintained improvements in dietary Phe tolerance over 3.5 years. These results continue to support the favourable risk/benefit profile for sapropterin in paediatric patients (< 4 years of age) with BH_4_-responsive PKU. Frequent monitoring of blood Phe levels and careful titration of dietary Phe intake to ensure adequate levels of protein intake is necessary to optimise the benefits of sapropterin treatment.

*Trial registration* ClinicalTrials.gov, NCT01376908. Registered 17 June 2011, https://clinicaltrials.gov/ct2/show/NCT01376908.

**Supplementary Information:**

The online version contains supplementary material available at 10.1186/s13023-021-01968-1.

## Background

Hyperphenylalaninaemia (HPA) is a rare, inherited metabolic disorder caused by variants in the gene encoding the enzyme phenylalanine hydroxylase (PAH), which catalyses the conversion of phenylalanine (Phe) to tyrosine (Tyr) [1]. HPA presents with a spectrum of phenotypes that can be grouped into one of the three main categories according to pre-treatment blood Phe concentrations: classical phenylketonuria (PKU; Phe ≥ 1200 μmol/L), mild PKU (Phe 600–1199 μmol/L), and mild HPA (Phe 120–599 μmol/L) [2–4]. The incidence of PKU in Europe is approximately 1:10,000 births [5], although higher rates are seen in countries such as Turkey (1:4192) [6] and Ireland (1:4500) [7]. Untreated or poorly managed PKU in paediatric patients is characterised by a range of symptoms, including intellectual disability, microcephaly, motor deficits, autism, seizures, developmental problems, neuropsychological symptoms, and skin problems (eczema) [5].

European guidelines for the treatment of PKU recommend a target blood Phe level of 120–360 μmol/L in patients < 12 years of age [5, 7]. Sapropterin dihydrochloride (sapropterin, Kuvan®, BioMarin, Novato, CA, USA) is a synthetic formulation of tetrahydrobiopterin (BH_4_), the naturally occurring cofactor of PAH, and was initially approved in Europe to reduce blood Phe levels, in conjunction with a Phe-restricted diet, in patients ≥ 4 years of age with BH_4_-responsive PKU [8].

The Safety Paediatric efficAcy phaRmacokinetics with Kuvan® (SPARK) study [9] (NCT01376908) was undertaken to assess the effect of sapropterin plus a Phe-restricted diet versus a Phe-restricted diet alone, on dietary Phe tolerance (the amount of dietary Phe (mg/kg/day) that can be consumed while maintaining blood Phe levels within the recommended range of 120–360 μmol/L) [5] in children < 4 years of age with BH_4_-responsive PKU or mild HPA. During the initial 26-week study period, the addition of sapropterin to a Phe-restricted diet led to a significant improvement in Phe tolerance versus a Phe-restricted diet alone (difference at 26 weeks: 30.5 mg/kg/day of prescribed Phe; 95% CI 18.7–42.3 mg/kg/day; *p* < 0.001). Furthermore, the observed safety profile of sapropterin was consistent with that seen in older patients [9]. Based on these results, the approved indication for sapropterin in Europe was expanded in 2015 to include patients < 4 years of age [10].

Herein, we present the results of the SPARK extension study (NCT01376908), which was designed to evaluate the long-term safety, dietary Phe tolerance, blood Phe levels and neurodevelopmental outcomes over an additional 36 months of treatment with sapropterin.

## Results

### Patient disposition

All 51 patients who completed the 26-week study period (patient disposition figure previously published) [9] entered the extension period. Of these, 33 patients (64.7%) completed the extension period (18 patients [72.0%] from the ‘sapropterin continuous’ group [those who were previously treated with sapropterin plus Phe-restricted diet] and 15 patients [57.7%] from the ‘sapropterin extension’ group [those who were previously treated with a Phe-restricted diet only]).

The remaining 18 patients (35.5%) discontinued the study prematurely; the reasons reported were ‘other’ (14 patients [77.8%]), ‘unknown’ (3 patients [16.7%]), and ‘lost to follow-up’ (1 patient [5.6%]). The majority of the discontinued patients were in the ‘other’ category and included the patients reaching an age where they could be taken off the study (as per protocol), patients switching to a commercial drug (as permitted by the protocol), or patients switching to another sapropterin study. Overall, 6 patients (11.8%; 4 patients from the ‘sapropterin continuous’ group and 2 patients from the ‘sapropterin extension’ group) had a major protocol deviation of ‘adherence to sapropterin < 80% or > 125%’ and were excluded from the PPE population (Additional file 1: Table S1).

### Patient demographics and clinical characteristics

The Intention-To-Treat Extension (ITTE) population for ‘sapropterin continuous’ and ‘sapropterin extension’ groups consisted of 25 and 26 patients, respectively; the corresponding Per Protocol Extension (PPE) population consisted of 21 and 24 patients, respectively. Treatment groups were balanced in all demographic characteristics (Table 1). The overall mean ± SD age was 19.9 ± 11.7 months (range 2–47 months). There were slightly more males than females in both groups (‘sapropterin continuous’ group: 60% vs. 40%; ‘sapropterin extension’ group: 53.8% vs. 46.2%). The patients were from Austria, Belgium, Czech Republic, Germany, Italy, Netherlands, Slovakia, Turkey, and the United Kingdom.

**Table 1:** Demographics and baseline characteristics (ITTE population)

Characteristics, statistics	‘sapropterin continuous’ (n = 25)	‘sapropterin extension’ (n = 26)
Age (months), mean (SD)	20.1 (12.1)	19.8 (11.5)
Age group, n (%)		
< 12 months	7 (28.0)	8 (30.8)
12 to < 24 months	9 (36.0)	8 (30.8)
24 to < 48 months	9 (36.0)	10 (38.5)
Female, n (%)	10 (40.0)	12 (46.2)
Race, n (%)		
White	24 (96.0)	25 (96.2)
Asian	0	1 (3.8)
Other	1 (4.0)	0
Height (cm), mean (SD)	81.21 (11.37)	80.73 (10.92)
Weight (kg), mean (SD)	11.14 (3.17)	11.09 (2.83)
Age at PKU diagnosis, days		
Mean (SD)	28.2 (83.0)	31.9 (75.5)
Min; Max	1; 425	4; 382
Blood Phe level at diagnosis (µmol/L), mean (SD)	738.9 (421.4)	788.4 (508.3)
Disease severity*		
Classical PKU, n (%)	4 (16.0)	5 (19.2)
Mild PKU, n (%)	9 (36.0)	7 (26.9)
MHP, n (%)	12 (48.0)	14 (53.8)

Note that in small children, even common childhood illnesses can cause temporary loss of metabolic control, which may lead a physician to stop dietary adjustments or to stop drug treatment. Such patients were excluded from analysis in the PPE population. Six patients (> 10%) moved from the PPE to ITTE population during the extension period

*ITTE* intention-to-treat extension (population), *MHP* mild hyperphenylalaninaemia, *Phe* phenylalanine, *PPE* per protocol extension (population), *PKU* phenylketonuria

^*^Defined as: MHP, 120–599 µmol/L Phe; mild PKU, 600–1199 µmol/L Phe; classical PKU, ≥ 1200 µmol/L Phe

### Dietary Phe tolerance after 36 months

**Fig. 1:**
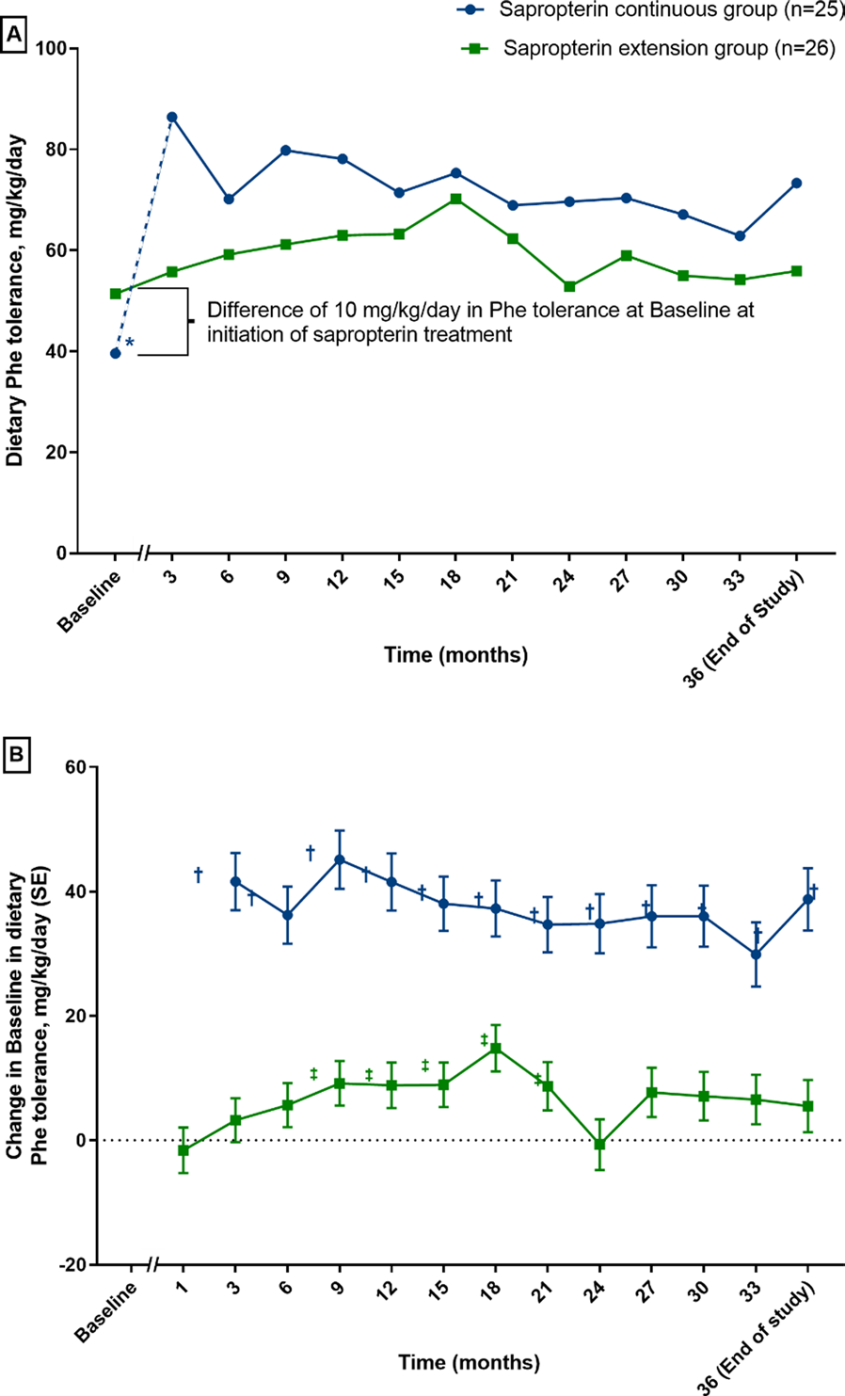
**a** Dietary Phe tolerance during the extension period. **b** Dietary Phe tolerance (mg/kg/day) change in baseline during the extension period. The Month 1 visit in the extension period was only for patients who entered the extension period and who had received only a Phe-restricted diet in the 26-week study period. *Baselines are not contemporaneous; for the ‘sapropterin continuous’ group, the baseline was 26 weeks prior to that for the ‘sapropterin extension’ group (indicated by the dotted blue line in **a**). ^†^*p* < 0.05 versus Baseline. ^‡^*p* < 0.001 versus Baseline. *Phe* phenylalanine

Dietary Phe tolerance increased significantly versus baseline, by 38.7 mg/kg/day at the end of the study in the ‘sapropterin continuous’ group (95% CI 28.9, 48.6; *p* < 0.0001; Fig. 1a, b) and significant increases were maintained throughout the 36-month duration of the study (Fig. 1b). In the ‘sapropterin extension’ group, a less pronounced effect was observed (Fig. 1a,b), with significant differences versus baseline only observed between months 9 and 21 (Fig. 1b). However, the analysis of the PPE population showed that the estimate of the difference compared to baseline was significant at the majority of time points, with the exception of months 3, 24, and 36 (end of Extension Period) only. Dietary Phe tolerance at the end of the study increased by 5.5 mg/kg/day versus baseline (95% CI − 2.8, 13.8; *p* = 0.1929). Importantly, the baselines were not contemporaneous. The baseline for the ‘sapropterin continuous’ group was the start date of sapropterin treatment in the 26-week study period (week 0), while for the ‘sapropterin extension’ group, baseline was the start date of sapropterin treatment within the extension period (week 27). If baselines for both groups were taken to be week 27, dietary Phe tolerance increased versus baseline, by 8.2 mg/kg/day in the ‘sapropterin continuous’ group. Notably, the Phe tolerance was 10 mg/kg/day higher at baseline (week 27) in the ‘sapropterin extension’ group compared with the baseline at week 0 in the ‘sapropterin continuous’ group (Fig. 1a).

### Blood Phe levels

**Fig. 2:**
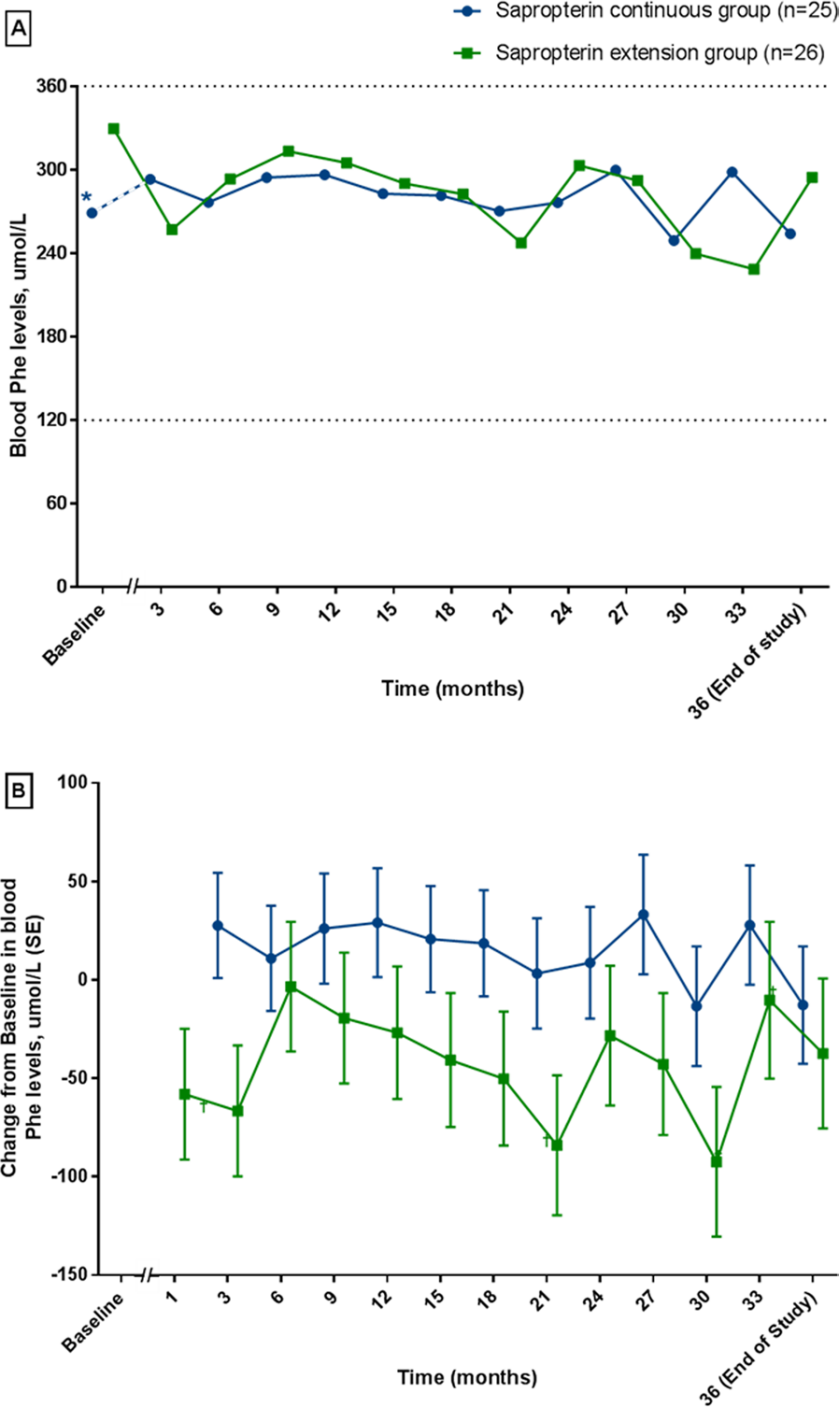
**a** Blood Phe levels during the extension period. **b** Blood Phe levels change in baseline during the extension period. The Month 1 visit in the extension period was only for patients who entered the extension period and who had received only a Phe-restricted diet in the 26-week study period. Range between dotted grey line indicates target range for blood Phe level. *Baselines are not contemporaneous; for the ‘sapropterin continuous’ group, the baseline was 26 weeks prior to that for the ‘sapropterin extension’ group (indicated by the dotted blue line in **a**). ^†^*p* < 0.05 versus Baseline. *Phe* phenylalanine, *SE* standard error

All patients maintained blood Phe levels within the guideline recommended range (120–360 μmol/L) during the extension period of the study (Fig. 2a). While blood Phe levels during the extension period in the ‘sapropterin continuous’ group remained stable over time, in the ‘sapropterin extension’ group, decreases in blood Phe levels versus baseline were observed at all visits, with statistically significant decreases observed at Months 21, 30 and 33 (Fig. 2b).

Treatment-emergent hypophenylalaninaemia was reported in a larger proportion of patients in the ‘sapropterin continuous’ group (11 patients [44.0%] with 45 events) compared with the ‘sapropterin extension’ group (3 patients [11.5%] with 6 events). However, hypophenylalaninaemia in this study was defined as blood Phe < 120 μmol/L, the upper limit of normal physiological levels of blood Phe. When a lower, more physiologically relevant definition of hypophenylalaninaemia was used (≥ 1 blood Phe level < 40 µmol/L) only 3 pateints in the ‘sapropterin continuous’ group and no patients in the ‘sapropterin extension’ group had blood Phe levels < 40 µmol/L during the study.

### Blood Tyr levels

No clinically relevant changes in blood Tyr levels were observed throughout the 3-year extension period. Tyr levels in the ‘sapropterin extension’ group were 68.2 (31.7) µmol/L at baseline and 54.8 (20.9) µmol/L at end of study; a change of − 18.1 (35.8) µmol/L. Tyr levels in the ‘sapropterin continuous’ group were 72.2 (38.1) µmol/L at baseline and 60.3 (31.3) µmol/L at end of study; a change of − 18.8 (45.0) µmol/L.

### Growth

During the 3-year extension period, weight standard deviation score (SDS), head circumference SDS and height SDS were considered to be normal in patients in both treatment groups with small, non-statistically significant changes in SDS noted from baseline (Table 2), suggesting normal growth velocity. Overall, body mass index SDS, height SDS and weight SDS remained stable for both treatment groups; changes from baseline to Month 36 for these SDS variables were small. For occipital-frontal head circumference, maximum increase from baseline was observed in the < 12-month age group at month 36 (mean change from baseline [SD]: ‘sapropterin continuous’ group, 7.95 [2.06] cm; ‘sapropterin extension’ group, 4.47 [0.98] cm).

**Table 2:** Change from baseline over time in growth parameters by age group (ITTE population)

Timepoint	‘sapropterin continuous’ (n=25)			‘sapropterin extension’ (n = 26)		
	Age					
	< 12 Months	12–< 24 Months	24–< 48 Months	< 12 Months	12–< 24 Months	24–< 48 Months
Body mass index SDS, mean (SD)						
Baseline value	− 0.39 (0.90)	0.56 (0.49)	0.78 (0.55)	0.26 (0.87)	0.70 (0.91)	0.30 (0.69)
Baseline—month 12	0.96 (0.91)	0.21 (0.94)	0.17 (0.42)	0.21 (0.68)	0.23 (0.80)	− 0.02 (0.50)
Baseline—month 24	0.46 (0.56)	− 0.03 (0.74)	− 0.16 (0.46)	0.47 (0.75)	− 0.11 (0.41)	− 0.23 (0.51)
Baseline—EOS	0.45 (0.91)	0.47 (0.74)	–	0.53 (0.89)	− 0.37 (0.07)	–
Head circumference (cm), mean (SD)*						
Baseline value	42.56 (2.52)	47.83 (2.25)	49.33 (2.42)	45.75 (1.89)	48.75 (2.92)	48.88 (1.65)
Baseline—month 12	6.09 (2.06)	1.66 (1.23)	1.33 (0.98)	2.96 (1.59)	1.31 (1.39)	0.93 (2.62)
Baseline—month 24	6.77 (2.44)	2.86 (0.80)	1.67 (1.03)	3.59 (1.01)	1.80 (1.10)	2.18 (1.61)
Baseline—EOS	7.95 (2.06)	3.54 (1.09)	2.68 (1.64)	4.47 (1.23)	1.36 (0.84)	2.63 (1.41)
Height SDS, mean (SD)						
Baseline value	0.43 (0.94)	− 0.28 (1.42)	− 0.49 (1.13)	0.11 (1.03)	− 0.19 (0.66)	− 0.35 (1.03)
Baseline—month 12	− 0.38 (0.58)	− 0.09 (0.80)	0.12 (0.66)	0.01 (0.65)	0.11 (0.48)	0.10 (0.57)
Baseline—month 24	− 0.46 (0.67)	0.28 (0.53)	− 0.37 (0.92)	− 0.16 (0.75)	0.19 (0.74)	0.11 (0.52)
Baseline—EOS	− 0.39 (0.81)	− 0.12 (0.62)	–	− 0.18 (0.55)	0.31 (0.62)	–
Weight SDS, mean (SD)						
Baseline value	− 0.04 (0.74)	0.21 (0.95)	0.22 (0.73)	0.25 (0.71)	0.38 (0.63)	− 0.01 (0.67)
Baseline—month 12	0.48 (0.48)	0.06 (0.60)	0.19 (0.31)	0.14 (0.36)	0.21 (0.38)	0.03 (0.25)
Baseline—month 24	0.10 (0.50)	− 0.01 (0.67)	− 0.34 (0.65)	0.22 (0.61)	0.01 (0.33)	− 0.10 (0.11)
Baseline—EOS	0.12 (0.34)	0.15 (0.83)	–	0.22 (0.66)	− 0.10 (0.39)	–

Treatment groups from the study period. Both groups received sapropterin + Phe-restricted diet in the extension period. Age group at study entry used

*EOS* end of extension period (month 36), *ITTE* intention-to-treat extension (population), *SD* standard deviation, *SDS* standard deviation score

^*^Measured as maximum occipital frontal head circumference

Growth parameters for the three patients who experienced blood Phe levels < 40 µmol/L were also suggestive of normal growth over the 3-year duration of the extension study.

### Neuromotor and neuropsychological development

The majority of patients had normal neuromotor development. No differences were observed between the groups for each development milestone (Table 3). All patients from the ‘sapropterin continuous’ and ‘sapropterin extension’ groups remaining in the study until Month 36 were assessed and showed normal development for personal-social skills. Development of fine and gross motor skills and language was also assessed to be normal in most patients in both groups by Month 36. When assessing the neurodevelopmental status, patients in both groups had stable mean (SD) values for each parameter throughout the study period (Table 4). Assessment of intelligence using Wechsler Preschool and Primary Scale of Intelligence in patients ≤ 42 months and > 42 months, respectively, at Years 1, 2 and 3 showed normal intelligence quotient (IQ) for patients in both study groups. At the end of the study, IQ scores were between 88.25 and 120.67 in both study groups, ranging around that of the general population (100) [11].

**Table 3:** Neuromotor developmental milestones (ITTE population)

Characteristics	Visit	Statistics	‘sapropterin continuous’ (n = 25)	‘sapropterin extension’ (n = 26)
Area of assessment fine motor skills, n (%)	Baseline	Normal	16 (69.6)	24 (92.3)
		Abnormal	7 (30.4)	2 (7.7)
	Month 12	Normal	16 (69.6)	22 (91.7)
		Abnormal	7 (30.4)	2 (8.3)
	Month 24	Normal	16 (94.1)	16 (88.9)
		Abnormal	1 (5.9)	2 (11.1)
	EOS	Normal	11 (84.6)	14 (100.0)
		Abnormal	2 (15.4)	0
Area of assessment gross motor skills, n (%)	Baseline	Normal	21 (91.3)	19 (73.1)
		Abnormal	2 (8.7)	7 (26.9)
	Month 12	Normal	21 (91.3)	22 (91.7)
		Abnormal	2 (8.7)	2 (8.3)
	Month 24	Normal	14 (82.4)	15 (83.3)
		Abnormal	3 (17.6)	3 (16.7)
	EOS	Normal	12 (92.3)	14 (100.0)
		Abnormal	1 (7.7)	0
Area of assessment language, n (%)	Baseline	Normal	20 (87.0)	22 (84.6)
		Abnormal	3 (13.0)	4 (15.4)
	Month 12	Normal	18 (78.3)	21 (87.5)
		Abnormal	5 (21.7)	3 (12.5)
	Month 24	Normal	14 (82.4)	14 (77.8)
		Abnormal	3 (17.6)	4 (22.2)
	EOS	Normal	12 (92.3)	11 (78.6)
		Abnormal	1 (7.7)	3 (21.4)
Area of assessment personal-social, n (%)	Baseline	Normal	20 (87.0)	20 (76.9)
		Abnormal	3 (13.0)	6 (23.1)
	Month 12	Normal	21 (91.3)	19 (79.2)
		Abnormal	2 (8.7)	5 (20.8)
	Month 24	Normal	16 (94.1)	14 (77.8)
		Abnormal	1 (5.9)	4 (22.2)
	EOS	Normal	13 (100.0)	14 (100.0)
		Abnormal	0	0

*EOS* end of extension period (month 36), *ITTE* intention-to-treat extension (population)

**Table 4:** Neurodevelopmental status assessment by age group (Wechsler preschool and primary scale of intelligence; ITTE population)

Timepoint	Statistics	‘sapropterin continuous’ (n=25)			‘sapropterin extension’ (n = 26)		
		Age					
		< 12 Months	12–< 24 Months	24–< 48 Months	< 12 Months	12–< 24 Months	24–< 48 Months
Full scale IQ (Wechsler scale of intelligence)							
Baseline	n (missing)	0 (7)	0 (9)	2 (7)	0 (8)	1 (9)	0 (8)
	Mean (SD)	–	–	115.50 (17.68)	–	124.00	–
Month 12	n (missing)	0 (7)	0 (9)	9 (0)	0 (8)	7 (3)	0 (8)
	Mean (SD)	–	–	110.44 (17.58)	–	111.43 (26.22)	–
Month 24	n (missing)	0 (7)	3 (6)	4 (5)	0 (8)	4 (6)	5 (3)
	Mean (SD)	–	98.00 (20.07)	105.50 (12.12)	–	115.25 (7.27)	94.40 (19.69)
EOS	n (missing)	6 (1)	1 (8)	4 (5)	5 (3)	5 (5)	4 (4)
	Mean (SD)	120.67 (14.67)	94.00	95.25 (8.26)	108.60 (14.81)	108.20 (14.75)	88.25 (14.17)
Performance IQ (Wechsler scale of intelligence)							
Baseline	n (missing)	0 (7)	0 (9)	2 (7)	0 (8)	1 (9)	0 (8)
	Mean (SD)	–	–		–	114.00	–
Month 12	n (missing)	0 (7)	0 (9)	9 (0)	0 (8)	7 (3)	0 (8)
	Mean (SD)	–	–		–	96.43 (20.26)	–
Month 24	n (missing)	0 (7)	3 (6)	4 (5)	0 (8)	4 (6)	5 (3)
	Mean (SD)	–	92.33 (22.01)	105.25 (4.99)	–	111.00 (12.19)	95.40 (22.79)
EOS	n (missing)	6 (1)	1 (8)	4 (5)	5 (3)	5 (5)	4 (4)
	Mean (SD)	116.33 (15.28)	90.00	93.75 (11.24)	96.80 (13.41)	103.40 (18.17)	89.75 (15.41)

Treatment Groups from the Study Period. Both groups received sapropterin + Phe-restricted diet in the extension period. Age group at study entry used. Missing could be either have been due to missing analysis values or age out of range for assessment

*EOS* end of study, *IQ* intelligence quotient, *ITTE* Intention-to-treat extension (population), *SD* standard deviation

### Safety

Overall, 96.1% of patients experienced at least one treatment-emergent adverse event (TEAE): all 25 patients in the ‘sapropterin continuous’ group and 24 of 26 patients in the ‘sapropterin extension’ group. Only 47 of 1401 TEAEs (3.4%) were assessed by the investigator as related to sapropterin (Table 5); the most commonly reported were ‘amino acid level decreased’ (hypophenylalaninaemia; 24 events occurring in 9 patients), amino acid level increased (4 events occurring in 2 patients), vomiting (4 events occurring in 3 patients), and rhinitis (3 events occurring in 3 patients).

**Table 5:** Overall summary of adverse events—Safety extension population

	‘sapropterin continuous’ (n = 25)		‘sapropterin extension’ (n = 26)		Overall (N = 51)	
	Patients n (%)	Events n (%)	Patients n (%)	Events n (%)	Patients n (%)	Events n (%)
TEAEs	25 (100)	838 (100)	24 (92.3)	563 (100)	49 (96.1)	1401 (100)
AEs related to sapropterin	9 (36.0)	40 (4.8)	4 (15.4)	7 (1.2)	13 (25.5)	47 (3.4)
SAE	6 (24.0)	12 (1.4)	7 (26.9)	7 (1.2)	13 (25.5)	19 (1.4)

*AE* adverse event, *SAE* serious adverse event, *TEAE* treatment-emergent adverse event

The proportion of patients who reported a serious adverse event (SAE) was similar between the treatment groups—6 patients (24.0%) with 12 events in the ‘sapropterin continuous’ group and 7 patients (26.9%) with 7 events in the ‘sapropterin extension’ group (Table 5). All SAEs were assessed as unrelated to sapropterin treatment. No patients withdrew from the study during the extension period due to an AE (Additional file 2: Table S2).

## Discussion

In patients with PKU, blood Phe concentrations need to be controlled as soon as possible after being diagnosed (normally detected via newborn screening programs) to prevent a range of symptoms, including intellectual disability, motor deficits, autism, seizures, developmental problems, behavioural issues and psychiatric symptoms and skin problems (eczema) [5]. Prior to 2015, as there was no licensed pharmacological treatment available in the EU, the only management option for children with PKU aged < 4 years was a Phe-restricted diet.

The initial 26-week study period of the SPARK trial showed that the addition of sapropterin to a Phe-restricted diet in patients < 4 years of age with BH_4_-responsive PKU or mild HPA significantly improved Phe tolerance, compared with a Phe-restricted diet alone [9]. These results are consistent with those seen in children 4–12 years of age treated with 20 mg/kg/day sapropterin, in whom the mean amount of Phe supplement tolerated had increased after 10 weeks of treatment [12].

The SPARK extension study was designed to evaluate the long-term safety, dietary Phe tolerance, blood Phe levels, and neurodevelopmental outcomes, over an additional 3 years of treatment with sapropterin. It demonstrated that long-term treatment with sapropterin plus a Phe-restricted diet in patients < 4 years of age maintains the increase in dietary Phe tolerance over the 3.5 year duration of the study, although a less pronounced effect was observed in the patients from the control arm of the 26-week study, who only initiated sapropterin treatment at week 27.

The effects on Phe tolerance were consistent with those reported in a study of similar length to the SPARK extension study; a study from the USA and Canada in children aged 0–6 years of age, in whom 20 mg/kg/day sapropterin treatment for 2 years lowered blood Phe concentrations, enabling, in some cases, an increase in dietary Phe intake [11].

The safety profile observed during the initial 26-week study period of SPARK [9] was confirmed during the 3-year extension period. The safety profile for sapropterin was acceptable and similar to that reported in patients < 4 years of age in the Phenylketonuria (PKU) Demographics, Outcomes and Safety (PKUDOS) registry in the USA [12].

Due to the young age of the children and the length of the study, all growth parameters were expected to increase substantially. To account for this, standard deviation scores were used to determine how close a child’s score is from the average mean for their age (given a value of 0).

During the 3-year extension period, weight SDS, head circumference SDS and height SDS were within normal growth parameters in patients in both treatment groups, indicating normal growth velocities in patients. Notably, despite improved Phe protein intake, no excessive weight gain was observed in this study. This might facilitate weight management in PKU patients, which is becoming an integral part of PKU treatment after studies have described an increased obesity risk in female patients with PKU, and its ensuing complications, as a possible consequence of dietary treatment [13–15].

Increase in dietary Phe tolerance was not significant at many time points in the ‘sapropterin extension’ group. However, the observed decreases in blood Phe levels in this group indicate that dietary Phe intake could have been increased further in these patients without exceeding the upper limit of recommended blood Phe levels, particularly if the algorithm used during the 26-week study period had also been followed in the extension period. The algorithm (alongside clinic visits every 2 weeks) was used to drive adjustments to dietary Phe intake during the 26-week study period, whereas in the extension period, dietary Phe intake was only adjusted at clinic visits every 3 months and in line with the practices of study centres (i.e. not driven by the algorithm). This disparity highlights the need for frequent monitoring of blood Phe levels in concert with careful titration of dietary Phe intake to ensure adequate levels of protein intake and to optimise the benefits of sapropterin treatment in patients < 4 years of age. It should be considered that the difference in Phe tolerance between the two groups may also in part be due to the type of mutation and hence phenotype. Additionally, differences in responsiveness of patients to sapropterin between the two groups may account for some of the disparity.

Mild to moderate degrees of growth impairment have been reported in children with PKU during dietary treatment [16], which may be due to insufficient protein intake or other dietary deficiencies. In the initial 26-week period of the SPARK study, the secondary endpoints of growth and neuromotor development were normal in the patient population throughout the study; however, the time scale was considered to be too short to expect clinically meaningful changes. The further 3 years of the SPARK extension study confirmed that growth and neuromotor development remained within normal parameters in this patient population treated with sapropterin plus a Phe-restricted diet during the entire study (‘sapropterin continuous’) or only in the extension period (‘sapropterin extension’).

Limitations of this study include differences between the initial 26-week study and the extension period, such as non-contemporaneous baselines, differences in the methods used to adjust dietary Phe intake (algorithm driven versus standard practice of the clinical centre), and adjustments to dietary Phe and/or sapropterin dose were performed less frequently in the extension period (every 3 months) compared with the 26-week study period (every 2 weeks). Patients with mild HPA, who comprised about half of the population in this study, retain substantial enzyme activity and will, therefore, likely respond to sapropterin treatment. However, the indication for treatment of mild HPA differs between countries due to a weak evidence base. Both US [7] (all ages) and European [5] (patients < 12 years of age) guidelines recommend treatment to lower blood Phe in patients with a Phe concentration above 360 μmol/L; however, these guidelines were not available at the time this study was initiated and, therefore, some countries may have only initiated treatment at Phe concentrations above 600 μmol/L [17].

In adults, effective Phe reduction is cited as that of 20–30% [7]. However, in children with PKU, no minimal clinically important differences (MCID) other than the Inattention subscale has been validated [18]. A MCID measure of Phe tolerance for children with PKU could be validated in future studies.

## Conclusions

Overall, long-term treatment with sapropterin plus a Phe-restricted diet in patients < 4 years old of age with BH_4_-responsive PKU or mild HPA maintained improvements in dietary Phe tolerance over 3.5 years, consistent with the results observed in studies undertaken in patients > 4 years of age and continue to support the favourable risk/benefit profile for sapropterin in paediatric patients (< 4 years of age) with BH_4_-responsive PKU. Frequent monitoring of blood Phe levels and careful titration of dietary Phe intake to ensure adequate levels of protein intake is necessary to optimise the benefits of sapropterin treatment.

## Methods

### Study description

The SPARK extension study is an open-label, multicentre, phase IIIb, 36-month extension of the initial 26-week randomised SPARK trial [9], designed to assess the efficacy and safety of sapropterin in patients < 4 years of age with BH_4_-responsive PKU or mild HPA. The study was conducted at 22 sites in nine countries (Austria, Belgium, Czech Republic, Germany, Italy, Netherlands, Slovakia, Turkey, and the UK).

The study was performed in accordance with the protocol (and subsequent protocol amendments) and with the ethical principles laid down in the Declaration of Helsinki, in accordance with the International Conference on Harmonisation (ICH), Note for Guidance on Good Clinical Practice (ICH Topic E6, 1996) and applicable regulatory requirements. The local ethics committee/institutional review board at each of the participating centres approved the protocol.

### Patients

As described in detail previously [9], male or female patients aged < 4 years of age at randomisation were eligible for entry into the SPARK study if they had a confirmed diagnosis of mild HPA or PKU, were responsive to BH_4_, had good adherence to dietary treatment, and either maintained blood Phe concentrations within the therapeutic target range (120–360 μmol/L) for 4 months prior to screening or had 75% of the last four values of Phe within the therapeutic range. Patients were excluded from the SPARK study if they had used sapropterin or BH_4_ within the previous 30 days, had known hypersensitivity to sapropterin or other formulations of BH_4_, or had a previous diagnosis of BH_4_ deficiency.

### Study design/treatments

In the initial 26-week study period of SPARK [9], patients needed to be responsive to BH_4_ (a decrease of > 30% in Phe concentrations following a 20 mg/kg BH_4_ challenge of at least 24 h). Patients were randomised 1:1 to receive 10 mg/kg/day sapropterin plus a Phe-restricted diet or a Phe-restricted diet only. Management of all patients was aimed at maintaining blood Phe levels within the recommended therapeutic range of 120–360 μmol/L through monitored dietary intake. After 4 weeks, if a patient’s Phe tolerance had not increased by > 20% versus baseline, the sapropterin dose could be increased to 20 mg/kg/day. Dietary Phe intake, blood Phe levels and adherence with sapropterin treatment were assessed every 2 weeks and dietary Phe intake was adjusted according to a set algorithm.

**Fig. 3:**
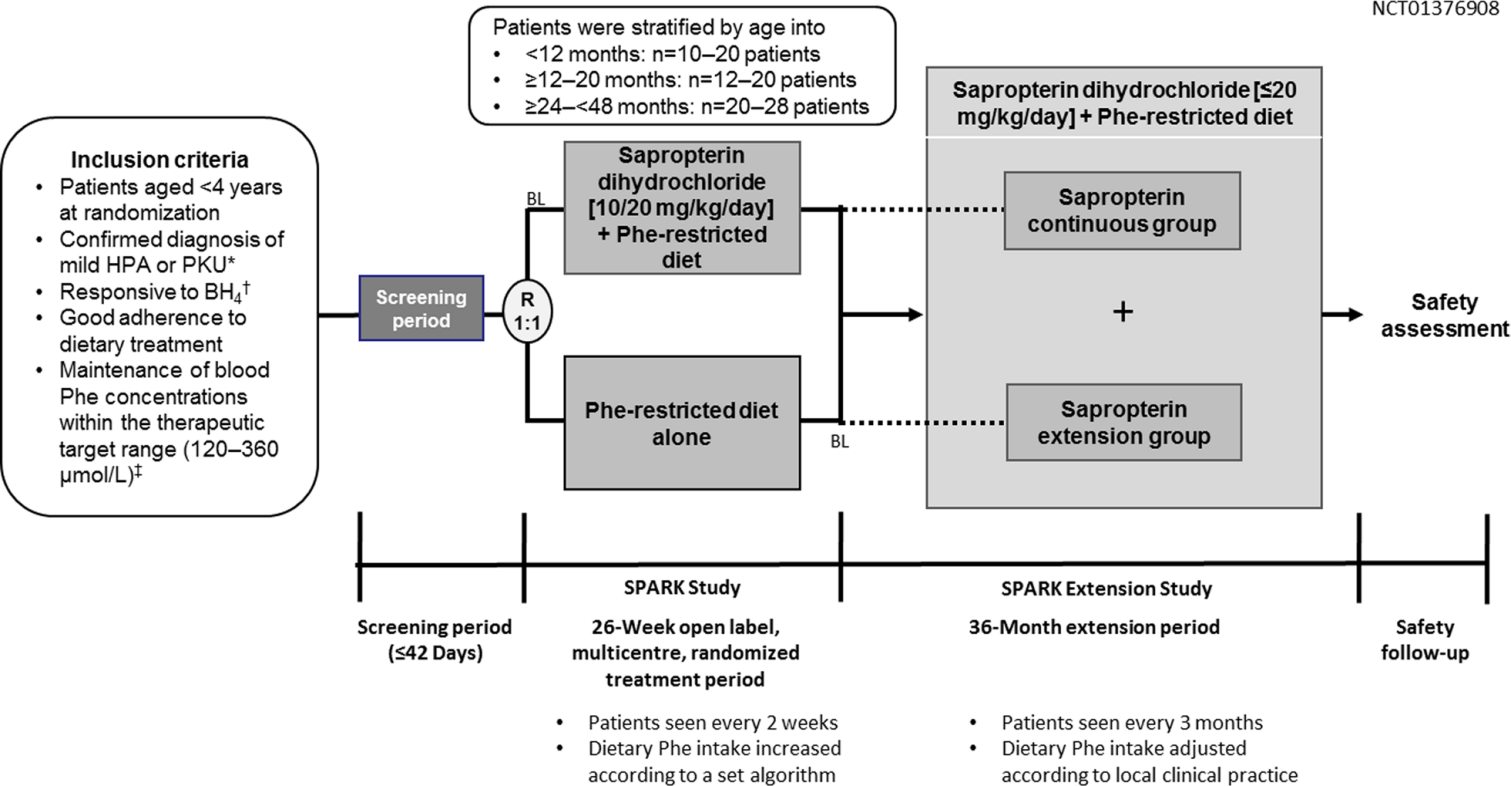
Study design. BL indicates the time point for baseline Phe levels for the ‘sapropterin continuous’ and ‘sapropterin extension’ groups in the SPARK Extension Study. BL was defined as the Day 1 of receiving sapropterin treatment in the 26-week SPARK study for the ‘sapropterin continuous’ group; for the ‘sapropterin extension’ group, baseline was defined as Day 1 of the SPARK Extension study, when patients started to receive sapropterin treatment on enrolment in the extension study. *A defined level of Phe tolerance consistent with a diagnosis of PKU, ≥ 2 previous blood Phe concentrations ≥ 400 μmol/L obtained on two separate occasions. ^†^BH_4_ responsiveness defined as a decrease of > 30% in Phe concentrations following a 20 mg/kg BH_4_ challenge of at least 24 h. Alternatively, 75% of the last four assessed values of Phe concentrations (either from venous blood or dry blood spot) should have been maintained in the therapeutic range. *BH*_*4*_ tetrahydrobiopterin, *BL* baseline, *HPA* hyperphenylalaninaemia, *Phe* phenylalanine, *PKU* phenylketonuria, *R* randomisation

After completing the initial 26-week study period, patients were eligible for enrolment in the SPARK extension study. Those who were previously treated with a Phe-restricted diet only, were initiated on sapropterin at 10 mg/kg/day, which could be increased up to 20 mg/kg/day (‘sapropterin extension’ group). Those who were previously treated with sapropterin plus Phe-restricted diet, remained on this regimen in the extension period (‘sapropterin continuous’ group) (Fig. 3). Baseline was defined as the start date of sapropterin treatment in the 26-week study period (week 0) for the ‘sapropterin continuous’ group and as the start date of sapropterin treatment within the extension period for the ‘sapropterin extension’ group (week 27). Dietary Phe intake, blood Phe levels, and adherence with sapropterin treatment were assessed every 3 months. Dietary Phe intake was adjusted according to clinical practice standards of each participating centre. Physical examination, growth parameters (height/length, weight, head circumference SDS [how many standard deviations an observation is above or below the mean age and gender-adjusted reference value for each measure from the World Health Organisation Growth Child standards reference]), and recording of AEs (particularly the incidence of hypophenylalaninaemia) were also undertaken every 3 months.

Blood Phe and Tyr levels, standard clinical laboratory measurements and neuromotor development milestones and status measurements were taken every 6 months. Standardised developmental milestones were assessed using a parent/guardian report form and neurodevelopmental status assessment was performed using the adaptive behaviour composite score with the standardised Bayley III Scales of Infant and Toddler Development for patients < 3.5 years of age and the social-emotional composite score in the WPPSI-III (Wechsler Preschool and Primary Scale of Intelligence) for patients ≥ 3.5 years of age.

Patients who turned 4 years of age during the extension period could remain in the study, or discontinue and receive commercial product. All patients underwent a standard safety assessment during a clinic visit 4 weeks’ post-treatment.

### Efficacy/safety assessments

The primary endpoint was dietary Phe tolerance. Secondary endpoints included neuromotor and neuropsychological development, and change in physical growth parameters. Safety analyses included the reporting and recording of AEs, including the severity and relationship to the study medication.

Efficacy analyses were performed in two populations: the ITTE, all patients randomised in the study period who continued in the extension period; and the PPE, all patients in the ITTE population without any major protocol deviations. The ITTE population is reported here, unless otherwise specified. The safety extension population consisted of all patients who received at least one dose of sapropterin and had some safety assessment data available (at least one visit for assessment of vital signs, AEs or laboratory results).

### Statistical analyses

Dietary Phe tolerance (mg/kg/day) was described using summary statistics at each time point of the extension period according to age group (< 12 months; ≥ 12 to < 24 months; ≥ 24 to < 48 months) and analysed using a linear mixed model for repeated measures on the observed records, applying the likelihood method. The model included the following fixed effects: time point, baseline dietary Phe tolerance, blood Phe level, age group and baseline mean blood Phe level. A covariance-variance matrix with a compound symmetry structure was used in the model in order to remain consistent with previous sapropterin studies [9]. The restricted maximum likelihood method was used and the degree of freedom was derived using the Kenward Roger computation [19].

Physical growth parameters were described using summary statistics, according to treatment and age group, and analysed using a linear mixed model for repeated measures to assess possible treatment effects. Neuromotor development was also described using summary statistics according to treatment and age group. Safety data was reported descriptively.

## Supplementary Information

**Additional file 1: Table S1.** Status at the end of the extension period—ITTE population.

**Additional file 2: Table S2.** Blood Phe levels in patients with treatment-emergent hypophenylalaninaemia.

## Data Availability

The datasets used and/or analysed during the current study are available from the corresponding author on reasonable request.
