# Supplementary material for: Long-term efficacy and safety of sapropterin in patients who initiated sapropterin at < 4 years of age with phenylketonuria: results of the 3-year extension of the SPARK open-label, multicentre, randomised phase IIIb trial
Source: Orphanet J Rare Dis. 2021 Aug 3;16:341. doi: 10.1186/s13023-021-01968-1 (PMC8335897; doi:10.1186/s13023-021-01968-1)
Supplement: Supplementary file 1 — Additional file 1: Table S1. Status at the end of the extension period—ITTE population. [file 13023_2021_1968_MOESM1_ESM.docx]

**Additional file 1: Table S1. Status at the end of the extension period – ITTE population**

| **Characteristics, statistics** | **‘sapropterin continuous’** | **‘sapropterin  extension’** | **Overall** |
| --- | --- | --- | --- |
|  | **(n=25)** | **(n=26)** | **(N=51)** |
| Status at the end of the extension period, n (%) |  |  |  |
| n (missing) | 25 (0) | 26 (0) | 51 (0) |
| Completed | 18 (72.0) | 15 (57.7) | 33 (64.7) |
| Discontinued prematurely | 7 (28.0) | 11 (42.3) | 18 (35.3) |
| Primary reason for early treatment termination, n (%) |  |  |  |
| n (missing) | 7 (0) | 11 (0) | 18 (0) |
| Lost to follow-up | 0 | 1 (9.1) | 1 (5.6) |
| Other* | 5 (71.4) | 9 (81.8) | 14 (77.8) |
| Unknown | 2 (28.6) | 1 (9.1) | 3 (16.7) |

*The majority of patients in this category discontinued during the extension period for the following reasons: Patient had reached an age where they could be taken off as per protocol, patient switched to commercial drug as allowed per protocol, or patient switched to another sapropterin study.

Note: Treatment Groups from the Study Period. Both groups received sapropterin + Phe-restricted diet in the extension period.

ITTE, Intention-To-Treat Extension (population).
