# Supplementary material for: Long-term efficacy and safety of sapropterin in patients who initiated sapropterin at < 4 years of age with phenylketonuria: results of the 3-year extension of the SPARK open-label, multicentre, randomised phase IIIb trial
Source: Orphanet J Rare Dis. 2021 Aug 3;16:341. doi: 10.1186/s13023-021-01968-1 (PMC8335897; doi:10.1186/s13023-021-01968-1)
Supplement: Supplementary file 2 — Additional file 2: Table S2. Blood Phe levels in patients with treatment-emergent hypophenylalaninaemia. [file 13023_2021_1968_MOESM2_ESM.docx]

**Additional file 2: Table S2. Blood Phe levels in patients with treatment-emergent hypophenylalaninaemia**

|  | | | **‘sapropterin continuous’  (n=25)** | | | | | | | | **‘sapropterin extension’ (n=26)** | | |
| --- | --- | --- | --- | --- | --- | --- | --- | --- | --- | --- | --- | --- | --- |
|  | | | **Patient number** | | | | | | | | | | |
| **Blood Phe µmol/L** | |  | **1** | **2** | **3** | **4** | **5** | **6** | **7** | **8** | **1** | **2** | **3** |
| **Initial 26-week study (Week)** | ***Screening*** | **F** | - | - | - | - | - | - | - | - | - | - | - |
|  |  | **V** | - | - | - | - | - | <50 | - | - | - | - | - |
|  | **Baseline** | **F** | - | - | - | - | - | - | - | - | - | - | - |
|  |  | **V** | - | - | - | - | 14 | - | - | - | - | - | - |
|  | **Day one** | **F** | - | - | - | - | - | 47 | - | **38** | - | - | - |
|  |  | **V** | - | - | - | - | - | - | - | - | - | - | - |
|  | **2** | **F** | - | 111 | - | - | - | - | - | - | - | - | - |
|  |  | **V** | - | - | - | - | - | - | - | - | - | - | - |
|  | **4** | **F** | - | 106 | - | - | - | - | - | - | - | - | - |
|  |  | **V** | - | - | - | - | - | - | - | - | - | - | - |
|  | **8** | **F** | - | - | - | - | - | - | 111 | - | - | - | - |
|  |  | **V** | - | - | - | - | - | - | - | - | - | - | - |
|  | **10** | **F** | - | - | - | - | - | - | 89 | - | - | - | - |
|  |  | **V** | - | - | - | - | - | - | - | - | - | - | - |
|  | **26** | **F** | - | - | - | - | - | - | - | - | - | - | - |
|  |  | **V** | - | - | **11** | 99 | 59 | - | 80 | 110 | - | - | - |
| **Extension period (Month)** | **Repeat screening** | **F** | - | - | - | - | - | - | - | - | - | - | - |
|  |  | **V** | - | - | - | - | - | - | - | - | - | - | 77 |
|  | **3** | **F** | - | - | - | - | - | - | 69 | - | - | - | - |
|  |  | **V** | - |  | - | - | - | - | - | - | - | - | - |
|  | **6** | **F** | - | - | - | - | - | - | 112 | - | - | - | - |
|  |  | **V** | 50 |  | **9** | - | - | - | 86 | - | 69 | - | - |
|  | **12** | **F** | - | - | - | - | - | - | - | - | - | - | - |
|  |  | **V** | - | - | 73 | 66 | - | - | - | - | - | - | 112 |
|  | **18** | **F** | - | - | - | - | - | - | 68 | - | - | - | - |
|  |  | **V** | - | - | - | - | 93 | - | - | - | 103 | - | - |
|  | **21** | **F** | - | - | - | - | - | - | - | - | - | 104 | - |
|  |  | **V** | - | - | - | - | - | - | - | - | - | - | - |
|  | **24** | **F** | - | - | - | - | - | - | - | - | - | 101 | - |
|  |  | **V** | - | - | - | - | - | - | 91 | - | - | 101 | - |
|  | **30** | **F** | - | - | - | - | - | - | - | - | - | 102 | - |
|  |  | **V** | - | - | - | - | - | - | - | - | - | - | - |
|  | **36** | **F** | - | - | - | - | 57 | - | - | - | - | - | - |
|  |  | **V** | - | - | 72 | - | **38** | - | - | - | - | - | - |
|  | **PT** | **F** | - | - | - | - | - | - | - | - | - | - | - |
|  |  | **V** | - | - | - | - | - | - | - | - | **31** | - | - |

Note: Treatment-emergent hypophenylalaninaemia is defined as any treatment-emergent adverse event that occurred after the first dose of sapropterin to the end of the study. For the ‘sapropterin continuous’ group, this was Day 1 in the Study Period and for the ‘sapropterin extension’ group, this was Day 1 in the Extension Period. F, filter paper; Phe, phenylalanine; PT, post-trial; V, venous.
